# FoxM1 is a promising candidate target in the treatment of breast cancer

**DOI:** 10.18632/oncotarget.23182

**Published:** 2017-12-12

**Authors:** Xiao-Feng Lu, De Zeng, Wei-Quan Liang, Chun-Fa Chen, Shu-Ming Sun, Hao-Yu Lin

**Affiliations:** ^1^ Department of Breast and Thyroid Surgery, The First Affiliated Hospital of Shantou University Medical College, Shantou, China; ^2^ Department of Medical Oncology, Cancer Hospital of Shantou University Medical College, Shantou, China

**Keywords:** FoxM1, breast cancer, prognostic values

## Abstract

Forkhead box protein M1(FoxM1) is a member of forkhead superfamily transcription factors. Emerging evidences have progressively contributed to our understanding on a central role of FoxM1 in human cancers. However, perspectives on the function of FoxM1 in breast cancer (BC) remain conflicting, and mostly were from basic research. Here, we explored the expression profile and prognostic values of FoxM1 based on analysis of pooled clinical datasets derived from online accessible databases, including *ONCOMINE*, Breast Cancer Gene-Expression Miner v4.0, and Kaplan-Meier plotter. It was found that, FoxM1 mRNA expression was significantly higher in breast tumor versus normal control. FoxM1expression profile presented a distinct pattern in different molecular subtypes of BC patients. Higher expression of FoxM1 was correlated to low mRNA expression of estrogen receptor 1 (ESR1), erb-B2 receptor tyrosine kinase 2 (ERBB2), and was inversely associated with the expression of classical luminal regulators forkhead box protein A1 (FoxA1) and GATA binding protein 3 (GATA3). Elevated FoxM1 expression predicted shorter distance metastasis free survival (DMFS) in BC patients, particularly with estrogen receptor (ER) positive and Luminal A, Luminal B subtypes of BC. More interestingly, elevated FoxM1 expression predicted poor survival in breast cancer patients, especially in the ER (+), progesterone receptor (PR) (+) subgroups and BC patients received adjuvant chemotherapy only or treated with tamoxifen only. These results implied that FoxM1 is an essential prognostic factor and promising candidate target in the treatment of breast cancer.

## INTRODUCTION

The mammalian transcription factor FoxM1 is dominantly overexpressed and plays critical role in tumorigenesis, proliferation, and metastasis, as well as drug resistance in a broad range of human cancer types, such as lung, gastric, and breast cancers [1, 2].

A plenty of studies, including an insight computational analysis, have demonstrated that elevated FoxM1 expression is a major predictor of adverse outcomes across a variety of human malignancies, indicating the oncogenic activity of FoxM1 in cancer. Recently, it was identified that FoxM1 acts as a critical regulator of mammary differentiation with significant implications for the development of aggressive breast cancers [3]. Elevated expression of FoxM1 in breast cancer correlates with an undifferentiated tumor phenotype and a negative clinical outcome. Abdeljaoued, S. and colleagues also reported that overexpression of FoxM1 is an adverse prognostic factor in male breast cancer [4].

More interestingly, accumulating evidences indicated that FoxM1 was significantly involved in drug resistance that compromised the efficacy of transtuzumab, tamoxifen and taxanes in the treatment of breast cancer. Furthermore, FoxM1-targeted therapy could effectively restrain tumor development of cancer [4–7]. There is increasing awareness that development of FoxM1 inhibitor is a promising strategy for breast cancer therapy.

Nevertheless, viewpoints on the role of FoxM1 in breast cancer were mostly from basic studies, and lacking support from clinical data. In the current study, we carried out a data-mining process in a variety of public databases with clinical information to evaluate the potent function and prognostic value of FoxM1 expression in breast cancer, with attempt of providing informative clues for future development of FoxM1-targeted therapy and prognostic prediction in breast cancer.

## RESULTS

### FoxM1was significantly overexpressed in breast cancer comparing with normal breast tissue

**Figure 1:**
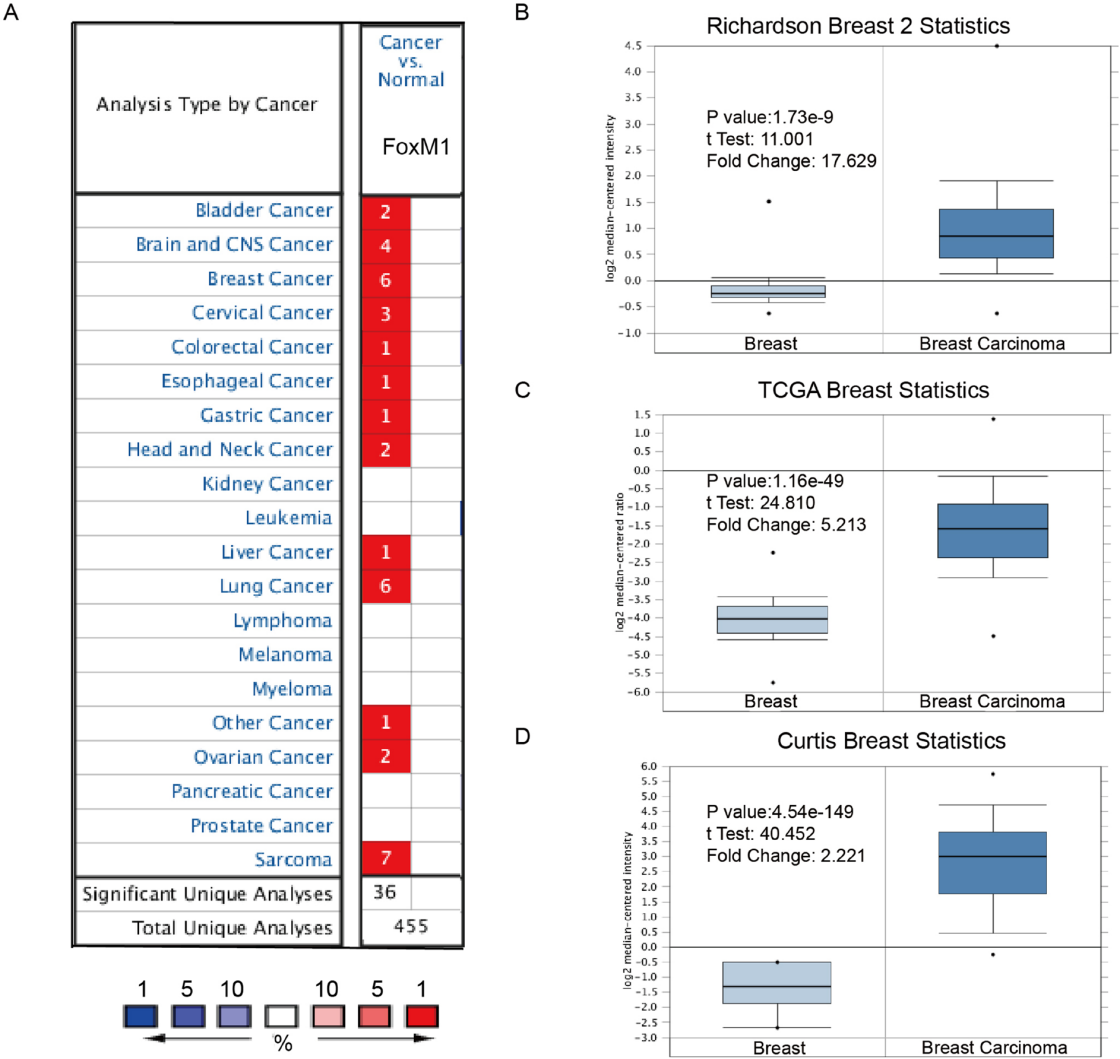
FoxM1 was significantly overexpressed in breast cancer comparing with normal breast tissue (**A**) FoxM1 mRNA expressions (cancer vs. normal tissue) analyzed with *ONCOMINE* database. The graphic demonstrated the numbers of datasets with statistically significant mRNA over-expression (red) or down-expression (blue) of the target gene. The *p* value threshold is 0.01. The number in each cell represents the number of analyses that meet the threshold within those analysis and cancer types. The gene rank was analyzed by percentile of target gene in the top of all genes measured in each research. Cell color is determined by the best gene rank percentile for the analyses within the cell. (**B**–**D**) Comparison of FoxM1 mRNA expression in Richardson’s study (B), TCGA breast statistics (C), and Curtis’s study (D). Box plots derived from gene expression data in *ONCOMINE* comparing expression of a specific GATA family member in normal and BC tissue. The *p* value was set up at 0.01 and fold change was defined as 2.

Hitherto, expression of FoxM1 had been identified in a number of human cancers, including hematological malignancies and solid tumors (Figure 1A). ONCOMINE analysis revealed that FoxM1 mRNA expression was significantly higher in a wide variety of datasets in different cancer types than corresponding normal samples, especially in sarcoma, lung cancer and breast cancer.

In a dataset from Richardson’s study [5], FoxM1 was 17.629-fold elevated in breast cancer samples as compared with normal tissues (*p* = 1.73e-9) (Figure 1B). Another dataset with 593 samples that derived from the Cancer Genome Atlas (TCGA) database showed that FoxM1 transcripts were 5.213-fold elevated in breast cancer samples as compared with normal tissues (Figure 1C). Consistently, in another dataset from Curtis’s study [6], FoxM1 was 2.221-fold increase in cancer VS. normal samples (*p* = 0.001).

### Expression of FoxM1 was distinct in different molecular subtypes of BC patients

**Figure 2:**
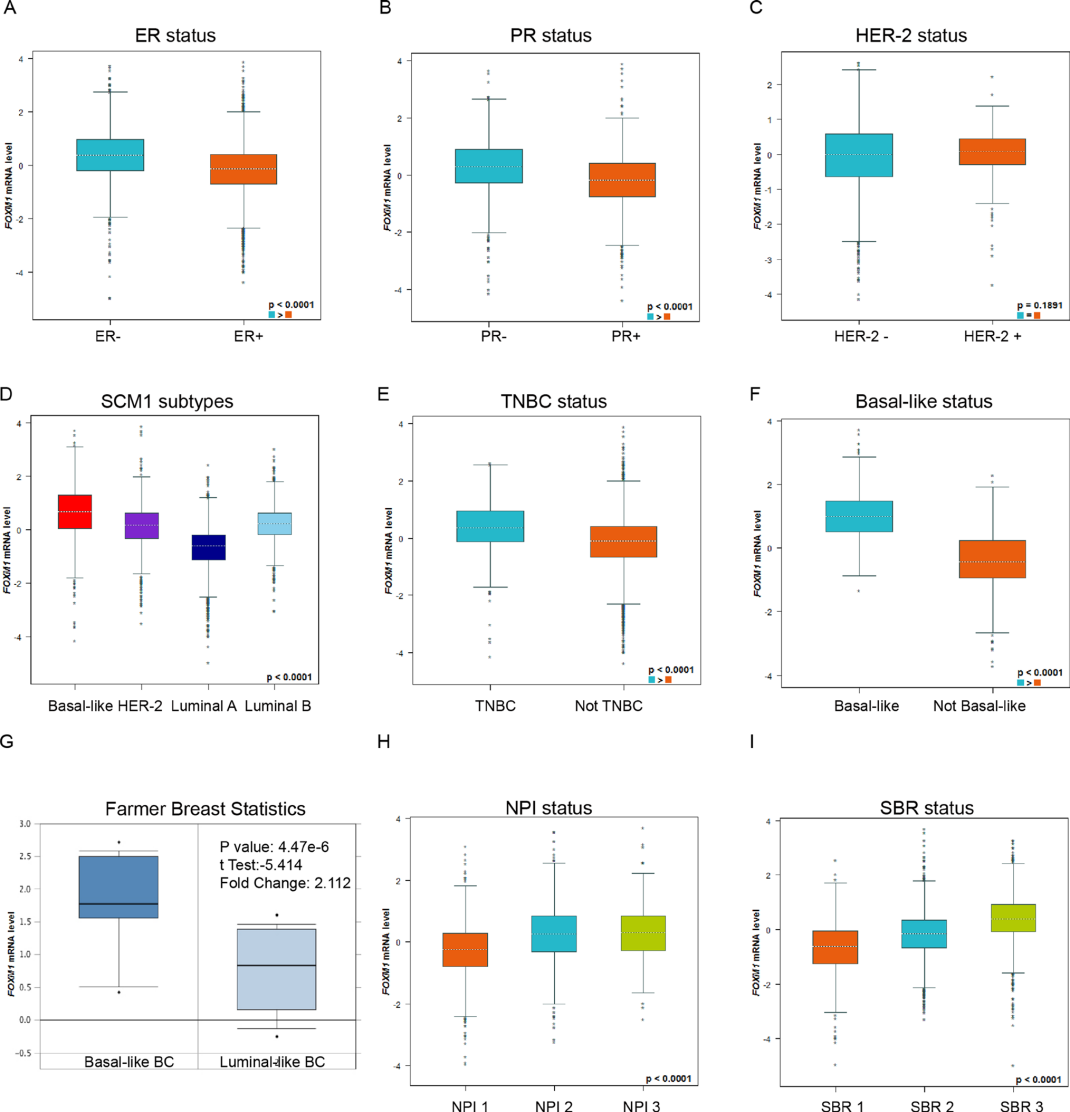
Expression of FoxM1 was distinct in different molecular subtypes of BC patients Global significant different between groups was assessed by Welch’s test to generate *p* value, along with Dunnett-Tukey-Kramer’s tests for pairwise comparison when a global significant difference exists (*p* < 0.05). (**A**) The mRNA expression level of FoxM1 in BC patients with ER (−) and ER (+). (**B**) The mRNA expression level of FoxM1 in BC patients with PR (−) and PR (+). (**C**) The mRNA expression level of FoxM1 in BC patients with HER-2 (−) and HER-2(+). (**D**) The mRNA expression level of FoxM1 in a variety of breast cancer subtype. (**E**) The mRNA expression level of FoxM1 in TNBC or NOT TNBC patients. (**F**) The mRNA expression level of FoxM1 in Basal-like or NOT Basal-like breast cancer subtype. (**G**) The mRNA expression level of FoxM1 in Basal-like or Luminal-like breast cancer subtype. (**H**) The mRNA expression level of FoxM1 in different Nottingham Prognostic Index(NPI) BC subgroups. (**I**) The mRNA expression level of FoxM1 in different Scarff Bloom & Richardson grade status (SBR) BC subgroups.

In bc-GenExMiner, the Welch’s test was performed to compare the mRNA expression of FoxM1 between groups of patients, according to different clinicopathological parameters. Higher FoxM1 mRNA levels were found in BC patients with ER (−) than ER (+) (Figure 2A). Similarly, increasing expression of FoxM1 mRNA was found in BC patients with PR (−) than PR (+) (Figure 2B). However, there was no significantly different between BC patients with HER-2 (+) and HER-2 (−) (Figure 2C).

For molecular subtypes analysis, the expression of FoxM1 in Basal-like and HER-2 subtypes was significantly higher than Luminal A and Luminal B subtypes of BC (Dunnett-Tukey-Kramer’s Tests, *p* < 0.0001) (Figure 2D, all of the groups comparison were showed in Supplementary Table 1). Consistently, higher FoxM1 mRNA levels were found in Basal-like or Triple-negative breast cancer (TNBC) patients (Figure 2E–2F). Another dataset from Farmer’s study [7] also showed that FoxM1 was 2.112-fold increase in Basal-like BC VS. Luminal-Like BC samples (*p* = 4.47e-6) (Figure 2G).

In Nottingham Prognostic Index (NPI), higher NPI level was associated with the enriched mRNA level of FoxM1 (Dunnett-Tukey-Kramer’s Tests, *p* < 0.0001) (Figure 2H, all of the group comparisons were showed in Supplementary Table 2). In Scarff Bloom & Richardson grade status (SBR) criterion, more advanced SBR grade was relevant to higher mRNA level of FoxM1 (Dunnett-Tukey-Kramer’s Tests, *p* < 0.0001) (Figure 2H, all of the group comparisons were showed in Supplementary Table 3).

### Higher expression of FoxM1 correlated with low expression of ESR1, ERBB2, FoxA1 and GATA3

**Figure 3:**
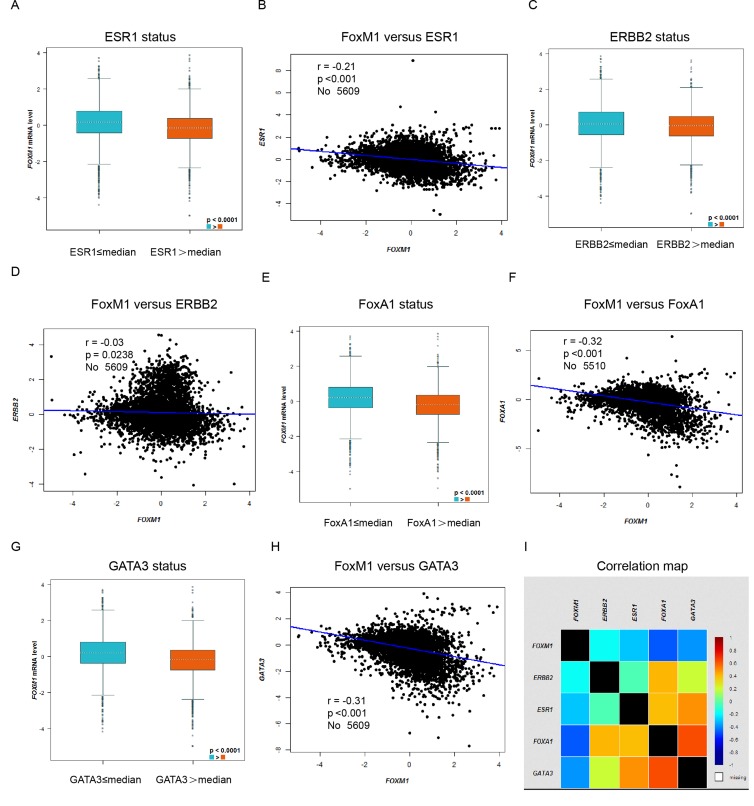
Higher expression of FoxM1 correlated with low expression of ESR1, ERBB2, FoxA1 and GATA3 In bc-GenExMiner, the Welch’s test was performed to compare the mRNA expression of FoxM1 between groups of patients, according to mRNA expression of different genes. (**A**) The mRNA expression level of FoxM1 according to mRNA expression level of ESR1. (**B**) Gene correlation targeted analysis between FoxM1 and ESR1. (**C**) The mRNA expression level of FoxM1 according to mRNA expression level of ERBB2. (**D**) Gene correlation targeted analysis between FoxM1 and ERBB2. (**E**) The mRNA expression level of FoxM1 according to mRNA expression level of FoxA1. (**F**) Gene correlation targeted analysis between FoxM1 and FoxA1. (**G**) The mRNA expression level of FoxM1 according to mRNA expression level of GATA3. (**H**) Gene correlation targeted analysis between FoxM1 and GATA3. (**I**) Correlation map for all patients among FoxM1, ESR1, ERBB2, FoxA1 and GATA3.

In bc-GenExMiner, the Welch’s test was performed to compare the mRNA expression of FoxM1 between groups of patients, according to mRNA expression of ESR1, ERBB2, and typical Luminal epithelial biomarkers FoxA1 and GATA3. Gene correlation targeted analysis showed that higher expression of FoxM1 correlated with low expression of ESR1 (Figure 3A–3B, *r* = −0.21, *p* < 0.001), ERBB2 (Figure 3C–3D, *r* = −0.03, *p* = 0.0238), FoxA1 (Figure 3E–3F, *r* = −0.32, *p* < 0.001) and GATA3 (Figure 3G–3H, *r* = –0.31, *p* < 0.001). Correlation map for all patients among FoxM1, ESR1, ERBB2, FoxA1 and GATA3 were showed (Figure 3I).

### Elevated FoxM1 expression predicted shorter DMFS in BC patients, especially in the ER positive and Luminal A, Luminal B subtypes of BC patients

**Figure 4:**
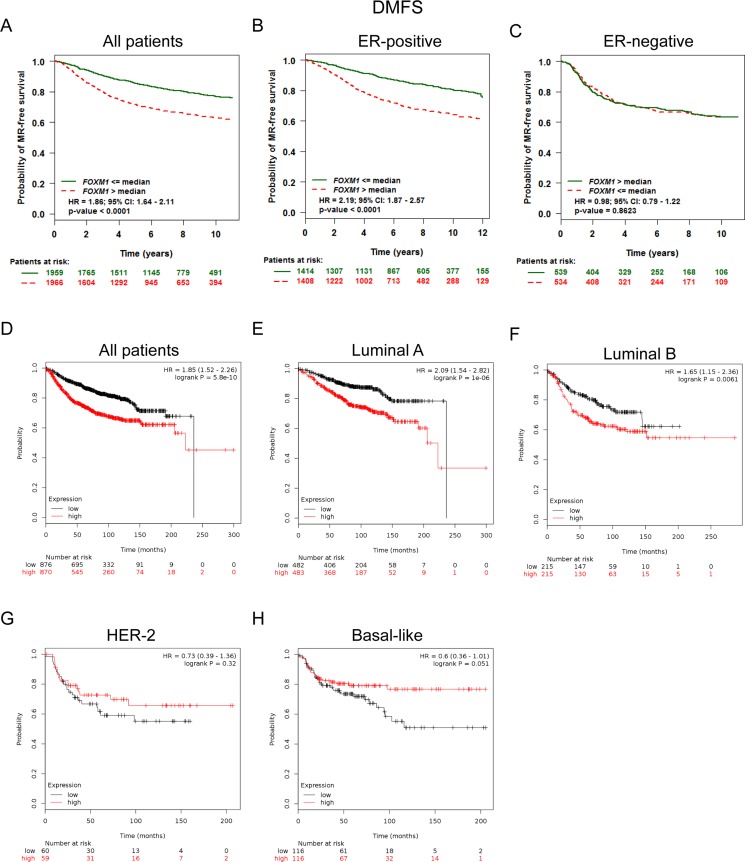
Elevated FoxM1 expression predicted shorter DMFS in BC patients, especially in the ER positive and Luminal A, Luminal B subtypes of BC patients (**A**) Analysis from bc-GenExMiner showed that FoxM1 mRNA expression was associated with DMFS in all BC patients (**B**–**C**) FoxM1 mRNA expression was correlated to DMFS in BC patients with ER positive tumors, but not in ER negative tumors. (**D**) The Kaplan-Meier plotter survival analysis showed that FoxM1 mRNA expression was correlated to DMFS in all BC patients. (**E**–**H**) The Kaplan-Meier plotter survival analysis showed that FoxM1 mRNA expression was correlated to DMFS in different subtypes of BC patients.

We next assessed the prognostic value of FoxM1 for distant metastasis in patients with BC. Analysis from bc-GenExMiner showed that FoxM1 mRNA high expression was associated with shorter DMFS in all BC patients (HR = 1.86, *p* < 0.0001) (Figure 4A). Sub-analysis indicated that FoxM1 mRNA high expression was correlated to shorter DMFS in BC patients with ER positive tumors (HR = 2.19, *p* < 0.0001) (Figure 4B), but not in ER negative tumors (HR = 0.98, *p* = 0.8623) (Figure 4C).

The Kaplan-Meier plotter survival analysis also showed that FoxM1 mRNA high expression was correlated to shorter DMFS in all BC patients (HR = 1.85, *p* = 5.8e–10) (Figure 4D), specifically in BC patients with Luminal A tumors (HR = 2.09, *p* = 1.0e–6) and Luminal B tumors (HR = 1.65, *p* = 0.0061) (Figure 4E–4F). However, there was no significant difference in DMFS in BC patients either with HER-2 positive (HR = 0.73, *p* = 0.32) tumors or Basal-like (HR = 0.6, *p* = 0.051) between high and low FoxM1 mRNA expression (Figure 4G–4H).

### Elevated FoxM1 expression predicted poor survival in breast cancer patients, especially in the ER (+), PR (+) subgroups and BC patients received adjuvant chemotherapy only or treated with tamoxifen only

**Figure 5:**
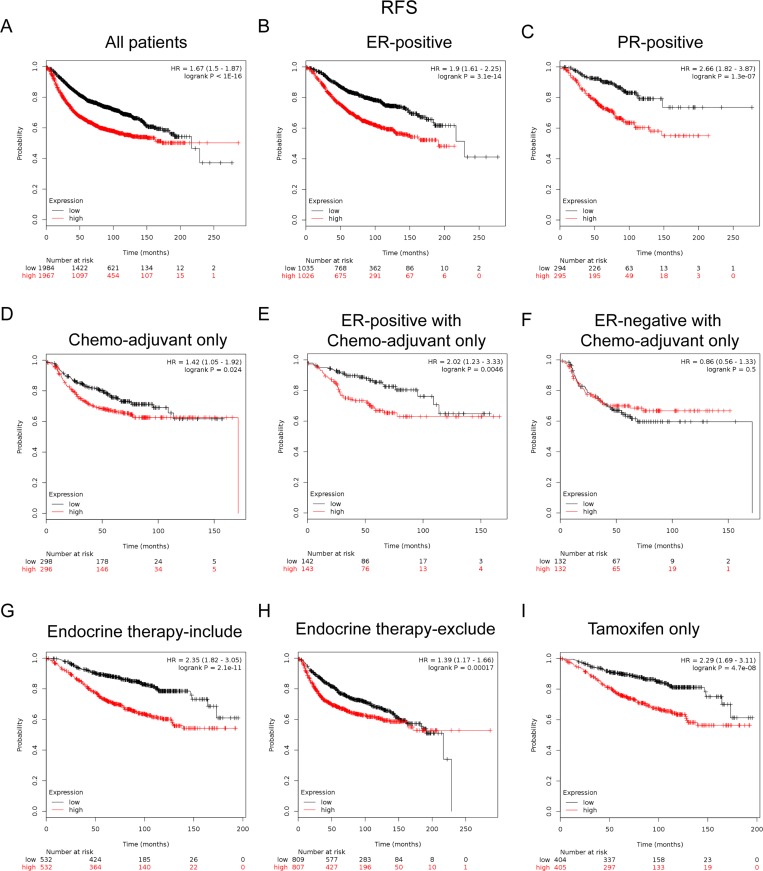
Elevated FoxM1 expression predicted poor survival in breast cancer patients, especially in the ER(+), PR(+) subgroups and BC patients received adjuvant chemotherapy only or treated with tamoxifen only (**A**) FoxM1 mRNA level was significantly associated with RFS in all BC patients. (**B**–**C**) FoxM1 mRNA expression was correlated to RFS in BC patients with ER (+) and PR (+) tumors. (**D**) High mRNA level of FoxM1 was significantly associated with shorter RFS in BC patients who have received chemotherapy adjuvant only. (**E**–**F**) High mRNA level of FoxM1 was significantly associated with shorter RFS in ER (+) subgroup of BC patients who have received chemotherapy adjuvant only but not in ER (−) BC patients. (**G**–**H**) High mRNA level of FoxM1 was significantly associated with shorter RFS in BC patients who have received therapies that include or exclude endocrine therapy. (**I**) High mRNA level of FoxM1 was associated with shorter RFS in BC patients who have received tamoxifen only sub-group.

FoxM1 mRNA high expression was significantly correlated with shorter RFS in all BC patients (HR = 1.67, *p* < 1.0e-16) (Figure 5A). FoxM1 mRNA high expression was correlated to shorter RFS in BC patients with ER positive tumors (HR = 1.9, *p* = 3.1e-13) (Figure 5B), and PR positive tumors (HR = 2.66, *p* = 1.3e-07) (Figure 5C).

Notably, for the respond to treatments, the results demonstrated that FoxM1 high mRNA expression was significantly correlated to shorter RFS in patients who have received adjuvant chemotherapy only (HR = 1.42, *p* = 0.024) (Figure 5D), especially in the ER-positive subgroup (HR = 2.02, *p* = 0.0046) (Figure 5E), but not in the ER-negative subgroup (HR = 0.86, *p* = 0.5) (Figure 5F). These results indicated a potential role of FoxM1 in contribution to chemoresistance in BC, which may even involve in the expression of ER.

Of noteworthy, the results demonstrated that FoxM1 high mRNA expression was significantly correlated to shorter RFS in patients who have received treatments included endocrine therapy (HR = 2.35, *p* = 2.1e–11) (Figure 5G) or excluded endocrine therapy (HR = 1.39, *p* = 0.00017) (Figure 5H), especially in patients who treated with tamoxifen only (HR = 2.29, *p* = 4.7e-8) (Figure 5I), indicating a potential role of FoxM1 in contribution to tamoxifen resistance in breast cancer.

## DISCUSSION

Discovery of therapeutic targets and development of novel targeted agents has changed the landscape in the battle field against breast cancer in the past few decades [8]. A variety of master genes, including ER, human epidermal growth factor receptor-2 (HER-2), and cyclin dependent kinase (CDK)4/6 have been identified to be involved in the development and progression of BC [9, 10]. Scientists have developed targeted agents against these genes or proteins that yielded successful results in the treatment of BC [11].

Myriad of studies have demonstrated that FoxM1 overexpressed in multiple cancers types [12–16], including breast cancer [4]. Recently, Li and colleagues provided a systematic review of prognostic value of FoxM1 in solid tumors, which demonstrated that elevated FoxM1 expression was associated with poor survival in most solid tumors. FoxM1 was a potential biomarker for prognosis prediction and a promising therapeutic target in human solid tumors [2]. Through analysis of a broad spectrum of pooled datasets, we found that FoxM1was significantly overexpressed in breast cancer comparing with normal breast tissue. Even more, the expression and prognostic value of FoxM1 in BC patients were highly associated with molecular subtypes and expressions of ER/PR, which imply the potentiality of FoxM1 acting as a master gene with important functions in breast cancer.

Several studies proposed that FoxM1 may promote distant metastasis through inducing of epithelial–mesenchymal transition (EMT) [17–19]. Xue and colleagues demonstrated that FoxM1 promoted typical EMT cellular pathway TGF-beta-dependent cancer metastasis via sustained activation of SMAD3/SMAD4 [20]. Wang and colleagues showed that increased FoxM1 expression is a target for metformin in the suppression of EMT in prostate cancer [21]. Yang and colleagues reported Slug to be the downstream target though which FoxM1 stimulating the processing of EMT [22]. Our data analysis agrees with those studies that elevated FoxM1 expression predicted shorter DMFS in BC patients, especially in patients with ER positive and Luminal A, Luminal B subtypes of BC. Also, our analysis on the correlation between FoxM1 and FoxA1, as well as GATA3, showed that FoxM1 gene was negatively associated with the classical luminal epithelial regulation gene GATA3 and FoxA1. These results indicated that FoxM1 was a negative regulator of breast epithelial phenotype. Therefore, FoxM1 might be a key driver and potential predictor of distant metastasis in BC patients.

More recently, increasing evidences have demonstrated a causal link between FoxM1 and chemoresistance. It has been reported that FoxM1expression associated with the chemotherapy resistance [23–29], especially in ER positive subgroup. Park and colleagues reported that FoxM1 mediates Dox resistance in breast cancer by enhancing DNA repair [30]. Khongkow and colleagues reported that paclitaxel targets FoxM1 to regulate KIF20A in mitotic catastrophe and dysfunction of FoxM1 expression will lead to breast cancer paclitaxel resistance [31]. Our analysis also indicated that elevated FoxM1 expression predicted worse survival in breast cancer patients with adjuvant chemotherapy only, particularly in the ER-positive subgroup. These results further confirmed the perspective that FoxM1 was a significant contributor of chemoresistance in breast cancer [32]. Although the exact mechanism are still unknown, some contributing reason could be self-explanatory. For example, the expression of FoxM1 in different breast cancer subtypes, as our data showed, was correlated to different expression patterns across different breast cancer subtypes. Moreover, the expression of FoxM1 was associated with tumor pathological grade. Particularly, high-level expressed in TNBC indicating that higher FoxM1 is an adverse prognostic factor and may involve in the chemoresistance of BC [33]. Evidence partly showed by our study suggested that FoxM1 was a pivotal driver of EMT, which was characterized with property of chemoresistance to BC. This perspective was supported by recent studies with results indicating that EMT contributes to chemoresistance of cancer [34, 35].

Of noteworthy, our results demonstrated that FoxM1 high mRNA expression was significantly correlated to shorter RFS in patients who have received endocrine therapy, especially in patients who treated with tamoxifen only, indicating a potential role of FoxM1 in contribution to tamoxifen resistance in breast cancer. Many studies revealed that high level of FoxM1 was associated with endocrine therapy sensitivity and resistance, especially tamoxifen resistance [36–39]. The possible reason is that the gene level FoxM1 is negatively related to the ESR1 expression, as our data showed, as well as negatively related to the ER expression in protein level. Genome-wide mapping of FoxM1 binding conducted by Sanders and colleagues also reveals co-binding of FoxM1 with estrogen receptor alpha in breast cancer cells [40]. Similarly, in our survival analysis, it was found that FoxM1 was associated with poor prognosis and early recurrence in patients with ER (+), PR (+), which implied that FoxM1 was actively involved in ER signaling pathway. This result agrees with the notion in the study by Millour and colleagues, proposing that FOXM1 is a transcriptional target of ER alpha and plays a critical role in breast cancer endocrine sensitivity and resistance [41].

A number of literatures reported that FoxM1was closely related to the expression of HER-2 [42, 43], and even some studies have documented that FoxM1has a direct control overHER-2 and may be the mediator of Herceptin resistance [44, 45]. Conversely, study by Francis, R and colleagues demonstrated that FoxM1was a downstream target and marker of HER-2 overexpression in breast cancer. However, our analysis showed that the correlation of FoxM1 and HER-2 expression remained uncertain. For one reason, the expression correlation coefficient *r* = –0.03 in the gene level of FoxM1 and ERBB2 gene is different from the perspective in an *in vitro* study by Kambach and colleagues [46]. Another reason is that the expression of FoxM1 in HER-2 (+) and HER-2 group (−) was not statistically difference. Finally, HER-2 subtypes in DMFS analysis of FoxM1 instead of the high expression of this seemingly contradictory phenomenon associated with good prognosis is warrant future research.

In conclusion, the oncogenic transcription factor FoxM1 is overexpressed in breast cancer versus normal controls. FoxM1 plays diverse roles in different molecular subtypes of breast cancer, which might be underlying the diverse mechanism of tumorigenesis and genetic background, as well as orchestrating with other cofactors in various tumor contexts. These evidences suggest that FoxM1 is an attractive prognostic prediction biomarker and promising therapeutic target for breast cancer.

## MATERIALS AND METHODS

### Oncomine analysis

The mRNA levels of FoxM1 in different type of cancers were determined through analysis in ONCOMINE database(www.oncomine.org), which is a publicly accessible online cancer microarray database to facilitate discovery from genome-wide expression analyses.

In this study, students’ *t*-test was used to generate a *p*-value for comparison between cancer specimens and normal control datasets. The fold change was defined as 2 and *p* value was set up at 0.01as described in our previous study [47]. Significant correlations can be found in an array of BC researches, as showed in typical figures.

### Breast Cancer Gene-Expression Miner v4.0 analysis

Breast Cancer Gene-Expression Miner v4.0 (bcGenExMiner v4.0) consisted 36 annotated genomic datasets and three statistical mining functions [48, 49]. The expression module was added on March 29th, 2016, comparing the expression of a target gene according to clinical criteria, such as hormonal receptors, nodal status, and so on. The prognostic module assessed the prognostic impact of candidate genes in human BC and provided potential prognostic markers for BC. The correlation module. computed the correlation between genes or identified clusters of correlated co-expressed genes located in the same chromosomal region.

### The Kaplan-Meier plotter survival analysis

Prognostic values of FoxM1was further assessed by displaying the relapse-free survival (RFS) and DMFS using the Kaplan-Meier plotter (www.kmplot.com). The log-rank p was calculated and shown on the webpage [50].

## SUPPLEMENTARY MATERIALS TABLES


