# Supplementary material for: FoxM1 is a promising candidate target in the treatment of breast cancer
Source: Oncotarget. 2017 Dec 12;9(1):842–52. doi: 10.18632/oncotarget.23182 (PMC5787517; doi:10.18632/oncotarget.23182)
Supplement: Supplementary file 1 [file oncotarget-09-842-s001.pdf]

FoxM1 is a promising candidate target in the treatment of breast cancer

SUPPLEMENTARY MATERIALS

Supplementary Table 1: The expression of FoxM1 in Basal-like and HER-2 subtypes was significantly higher than Luminal A and Luminal B subtypes of BC

Groups comparison	*p*-value*
HER-2 < Basal-like	$p < 0.0001$
Luminal A < Basal-like	$p < 0.0001$
Luminal B < Basal-like	$p < 0.0001$
Luminal A < HER-2	$p < 0.0001$
Luminal A < Luminal B	$p < 0.0001$
HER-2 < Luminal B	$p < 0.01$

*Dunnett-Tukey-Kramer's Tests, *p*(Welch) < 0.0001.

Supplementary Table 2: Higher nottingham prognostic index (NPI) level was associated with the enriched mRNA level of FoxM1

Groups comparison	*p*-value*
NPI2 > NPI1	$p < 0.0001$
NPI3 > NPI1	$p < 0.0001$
NPI3 = NPI2	$p > 0.10$

*Dunnett-Tukey-Kramer's Tests, *p*(Welch) < 0.0001.

Supplementary Table 3: Higher scarff bloom and richardson grade status (SBR) grade was associated with the enriched mRNA level of FoxM1

Groups comparison	*p*-value*
SBR2 > SBR1	$p < 0.0001$
SBR3 > SBR1	$p < 0.0001$
SBR3 > SBR2	$p < 0.0001$

*Dunnett-Tukey-Kramer's Tests, *p*(Welch) < 0.0001.
